# Exploring the benefits of participatory action research to a participatory data stewardship community project: the Round ‘Ere case study on data and well-being

**DOI:** 10.3389/fdgth.2025.1520825

**Published:** 2025-04-16

**Authors:** Emily S. Rempel, Gianfranco Polizzi, Simeon Yates

**Affiliations:** ^1^ Institute of Population Health, University of Liverpool, Liverpool, United Kingdom; ^2^ Department of Communication and Media, University of Liverpool, Liverpool, United Kingdom

**Keywords:** participatory research, data stewardship, datafication, public participation, qualitative research, well-being

## Abstract

**Introduction:**

Traditional data and measures about health and well-being provide vital insights but do not provide context on the ways in which a community may want to see development in their local area. This article is based on a Participatory Action Research (PAR) project on well-being and data conducted with members of a community in Widnes, a town in the UK. We explore the usefulness of adapting a PAR methodology to develop a Participatory Data Stewardship (PDS) program at the community level.

**Methods:**

Through repeated, semi-structured interviews, we tracked 15 Community Researchers' (CRs') experiences and perspectives of taking part in a PDS/PAR project. CRs were purposely recruited to primarily maximize diversity in gender, age, and socio-economic status, and interviewed before training, after training, and after fieldwork. We used thematic analysis to explore benefits and challenges, along with their expectations and experiences, at each stage of the project.

**Results:**

Four main themes emerged from interviews with CRs on their expectations and experiences: (1) the role of CRs' motivation in taking part on their perceptions of project impact, (2) the role and development of confidence in CRs' perceptions of their own success, (3) the importance of community building through an appreciation of diversity, and (4) the value in developing CR agency by putting participatory process at each stage of the project.

**Discussion:**

The findings illustrate that taking a PAR approach to the design of a PDS project around well-being and data shows potential for problematizing datafication through engaging local communities, developing their research skills, confidence and agency, and designing a data system that can empower community voice. This article addresses a gap in the literature on the feasibility of taking a PAR approach to the implementation of PDS. Future research should build on this study to explore the conditions for successful PAR in the context of other PDS projects.

## 1 Introduction

This interdisciplinary paper is based on a Participatory Action Research (PAR) and Participatory Data Stewardship (PDS) project conducted within Widnes, a town in the Liverpool City Region in the UK. Members of the community—here and throughout referred to as *Community Researchers* (CRs)—were trained in social science research methods to collect data from other residents about their perceptions of well-being and the role of data in making decisions to improve their community. We argue that such an approach was valuable for developing confidence and community building among CRs in Widnes, while also enabling them to exercise collective agency and acquire skills that may be useful beyond the project. This section presents a critical overview of relevant literatures and the research questions of this study. We briefly outline literature around datafication and data studies, participatory data stewardship, and public participation.

### 1.1 Dual narratives of datafication and the need for data justice in community

Datafication refers to how social life increasingly includes and relies on data where systems and institutions operate through the imagination, collection, and use of data (1, 2). The problem is that, for the large part, we live in societies where the application of data and policy decisions that are based on data are done *to* citizens rather than *with* citizens (3, 4). Datafication is often driven by corporate profit through the collection and sale of highly specific data about individuals, communities, and organizations (5). Those in positions of power, including government and digital and technology companies then benefit from datafication through this profit-making, despite the risks that this presents to individuals and communities. Individuals then bear the risks of these data decisions, as well as those presented by technologies, with minimal power to resist or address their use.

Risks of datafication range across issues including invasion of privacy, algorithmic bias, voter manipulation, denial of access to public services, and more (6, 7). A now infamous example of datafication risk can be found in the context of elections, as in the case of the Cambridge Analytica scandal, where massive secondary datasets collected through individual interaction with social media were (mis)used to produce target advertising designed through algorithmic processes to influence voting behavior (8). At the community and individual level, algorithmic bias can mean incorrect risk assessments leading to poor quality health care, longer jail sentences, and ineffective medical devices (9–11). When people, government, and organizations use data-driven algorithms in ways that reinforce discrimination or inequalities, communities are those who pay the price.

On the other hand, datafication is also driven by strong narratives around the potential of digital technologies to drive social change and derive community benefit, particularly from a government perspective (12, 13). Benefits can range from individual improvement in health and well-being to collective opportunities for research and community development (14–16). At the individual level, data derived from datafication has, for example, revolutionized care for Type 1 and 2 Diabetes through better modelling of daily glucose fluctuations (14). At the same time, more informative data sources can, at the community and city level, offer a range of benefits. These may include, for example, identifying food poverty using commercial data, helping address energy management with an aim of reducing reliance on fossil fuels, or using CCTV data to identify better locations for bike routes (15–17). In the context of this study, better data and information could support communities to target interventions, site services, and better represent lived realities to improve individual and community well-being. When people, government, and organizations use data-driven algorithms in ways that support different groups and promote equitable social change, communities can benefit.

While these dual narratives could sit in conflict, ultimately, they demonstrate that digital and data technologies do not intrinsically benefit all people, communities, and situations. This has led many scholars to call for the need for “data justice”, or specifically for a critical examination of the interaction between power, data, and social justice (7, 18, 19). Under a data justice framework, the field of critical data feminism argues that data research and technology can promote social justice while also addressing harm and risk—for example, through research advocating for the better tracking of feminicide (20, 21). D'Ignazio and Klein (20) argue for data science hegemonies to be challenged through explorations of power and context. However, *how* to challenge data science hegemonies at the community level is poorly understood. There is a paucity of research on the development and critical examination of methodologies on how data benefits can be promoted and harms addressed from datafication in community. Namely, this includes limited knowledge of what methods can support a community to ensure data-driven algorithms are used and deployed in line with principles of equity and social justice. In this paper, we problematize the notion of well-being data from this perspective. We designed and conducted a project that tested how PAR and PDS could be used to find opportunities to use data to improve communities in the recognition that (1) data use can be positive, and (2) mechanisms are needed that enable communities to use data in this way.

### 1.2 Defining, comparing, and contrasting PAR and PDS

This article deals with the concept of participation as twofold: in relation to (1) PDS and (2) the adoption of PAR, with a focus on the use of data to improve well-being. Well-being is used here as a multidimensional concept relating to the state of feeling physically, mentally, emotionally and socially healthy and prosperous, both from an individual perspective and, in the context of a community, in collective terms (22, 23). Meanwhile, data can be broadly defined as information that is imagined, collected, processed, or analyzed for specific purposes, including for making decisions about a particular issue (2, 4). PDS describes a model for public interaction with data systems and includes a range of “practices that empower people to help inform, shape and—in some instances—govern their own data” (3). This model relates to power and the question of who is (or is not) included in data processes. Proposed as one potential solution to the gap between imagined and realized benefit from data innovation, PDS is grounded in the objective of expanding public participation with data innovation, and therefore who benefits, through addressing power. However, there is a dearth of academic research on *how* PDS can deliver this potential and at what scale (3, 24). This article explores the adoption of PAR as a methodology for testing how PDS works in practice and specifically at the community level.

The concept of PDS draws on Shelley Arnstein's classic model of public participation demonstrating different intensities of public decision-making and community action around a political topic (25). Mapped to data systems, Patel (3) avoids seeing PDS as a ladder. Rather, she positions it as a spectrum of activities from informing communities on how data is used to empowering public participation in how data systems are designed, used, and governed, all of which are essential components to addressing datafication risk (3). Examples of what could be called PDS range immensely in form, function, and the degree to which they represent unheard voices in data. On the one hand, there are examples of data campaigning like the “Data Save Lives” initiative that emphasizes the potential for data to do good in health care and ensure individuals do not opt out of health data sharing initiatives (26). On the other end of the spectrum is the establishment of data trusts and data cooperatives (27–29). These projects propose using trusted intermediaries and collective action to create financial and social benefit for the individuals who produce data (27). As PDS emerges as a field of practice and study, what can be classified or reclassified as PDS is debatable. However, the core elements of a PDS project include addressing disempowered community and stewarding how and in what ways data can be imagined, collected, and used.

Like PDS, PAR shares an emphasis on participation as a methodology that is intended to empower members of a community to actively take part in one or multiple stages of a research project with an aim to create positive social change (30). Within health and well-being research, PAR is often used to address imbalances in power within healthcare systems to allow patients and community to influence the research process (30–32). Both PAR and PDS describe, and in many cases advocate for, the redistribution of power from a select few to a broader community through participatory mechanisms. Therefore, PAR's methodological underpinnings are potentially well-suited to taking a data justice approach to PDS emphasizing social change and participation as defining aspects. However, there is a paucity of research on the intersection of PDS and PAR for data justice– i.e., how a PAR methodology can be used, and with what benefits and implications there are to communities, in the context of a PDS project. Nevertheless, there are some broader community-based participatory data projects that can offer insights into the relevance of this intersection.

Relevant to exploring the design and usefulness of PAR to PDS is the implicit or explicit role of data in the project and the importance of considering power *within* a community in designing community data projects. A few studies have shown that data projects are often motivated by and designed to address complex factors like health, well-being, equity, engagement, and trustworthy government through the use of data (24, 33–38). For example, projects often engage both on the topic of data and on secondary topics like wellness (33), where the secondary topic is emphasized to encourage participation. These secondary topics, where data is implicitly discussed, are also a recognition of the complexity of how data fits into, and reflects, day-to-day life alongside existing social and political systems. Taylor et al.'s (34) and Lindley et al.'s (36) work on data-in-place in a UK context highlights that “byproduct” data, e.g., data created through datafication processes, is closely interwoven with the functioning and environment of place. The numbers and statistics used to represent a community will have emotions and history attached to what they represent. Therefore, when talking about data in community, it is a conversation about data and community, and the issues that community face.

When designing community data projects, organizers will often need to find a balance between the practical elements of recruiting community participants and diversifying who is represented (36, 39). Evidently, the people who have the most time and resources to get involved are usually the ones most likely to do so, i.e., community members that are represented in public engagement may already be in relative positions of power because they are able to navigate the processes of recruitment and participation (39). Within the context of participatory data, this has led to conflicts in engaging a wider and diverse group of people in a community (35, 36). Participatory methods that do not challenge power nor diversify who has a say in a datafied world would not address a core aim of participatory data stewardship, e.g., empowering people to shape data processes. This highlights an important gap in the literature in the need for critical reflection on the methodologies of PDS and their potential for challenging power in and between communities.

The themes and projects highlighted above also demonstrate the necessity of understanding community experiences of community data projects. Community perspectives are vital to fully evaluating the potential usefulness of PAR to PDS and of PDS in empowering community in the context of data justice. PAR offers an opportunity to explore and include community perspectives throughout the design of a PDS project. Due to their similar ideological and methodological grounding, PAR offers a unique lens through which to explore the feasibility of PDS and ultimately data justice at the community level.

### 1.3 Research questions

This paper addresses one main research question on the relationship between PAR and PDS:

RQ1: What is the usefulness of adapting a PAR methodology to develop a PDS program at the community level?

This article answers the question above through exploring the views and experiences of those taking part in such a program, thus addressing an additional question:

RQ2: What are the views and experiences of community members taking part in the different stages of a PAR project on data at the community level?

## 2 Materials and methods

Through repeated, semi-structured interviews, the research team (consisting of the authors of this article) tracked 14 CRs' experiences and perspectives of taking part in a PDS/PAR project called Round ‘Ere. Semi-structured interviews were conducted by the research team with CRs at three different points in time (before the training, after the training, and after the fieldwork). This article presents key findings from the interviews with CRs with a view to reflecting on the importance and feasibility of taking a PAR approach to community-based PDS. In this section, we describe the design of this project, our methodology, and analytical procedures. This project was approved by the University of Liverpool Research Ethics Committee—reference numbers: 11798 and 12124.

### 2.1 Designing the Round ‘Ere project

The PAR/PDS project, Round ‘Ere, involved working with a place-based community (Widnes, UK) to co-design and run a project on what feeling well meant to residents in their community with the aim of designing a community data hub that represented resident preferences and perspectives on well-being data. The community of Widnes where this study took place is defined by the UK Government as a “left behind” neighborhood (40). This was a metric used by government to site additional funding to regions in the wake of the COVID-19 pandemic (33, 40) and highlights issues around socioeconomic status and deprivation. Funded by the Liverpool City Region Combined Authority (LCRCA), Round ‘Ere was a collaboration between the Liverpool City Region Civic Data Cooperative (LCR CDC) (a data stewardship research project), Capacity (a Liverpool-based policy design company), and One Halton (a partnership between the NHS, Halton Borough Council, and the voluntary sector). LCR CDC and Capacity designed and ran the project, while One Halton were the main place-based stakeholder. We describe the project below, in brief, with further details available in the final Round ‘Ere report (41).

The project was designed with an appreciative inquiry approach (33)—specifically asking what beneficial uses of data and data technology could look like for a local community. The objective was to first ask what data and data technology could address in a community, and then to design a data hub or platform that meets the specifications and needs of the community. We use the term data “hub” or “platform” to represent a digital technology that links, categorizes, and makes actionable data (42).

This project forms part of a larger LCR CDC project that aims to make health and well-being data about the City Region work for the people who live there. LCR CDC is funded by the LCRCA and hosted at the University of Liverpool.

To this aim, Round ‘Ere, overall, was designed with three phases:

1. Community-led PAR on a health topic (to define datasets and potential projects)
2. Community data hub development (to represent community preferences for data collection and linkage)
3. Data hub testing through topic-based case studies (to run community-based data projects using the data hub)

This paper concerns itself specifically with the first phase of the Round ‘Ere project, namely designing a PAR project on a health topic with an aim of stewarding the direction of data collection, linkage, and technology development on that topic. We chose well-being as the health topic due to its holistic scope and ease of understanding for a lay audience, i.e., well-being is relevant for all residents, who do not need any specialist knowledge to discuss their own well-being and the well-being of their community. In the design of the project, we explored the concept of well-being data from an academic perspective to understand what kinds of data are currently collected and how they are used. At the same time, we drew on lessons learned in other similar place-based data projects both in the grey and academic literature, as reviewed earlier in this article.

In Spring 2023, we recruited and trained 16 CRs in listening skills, research methods, and data concepts, with 14 CRs finishing the project. CRs were purposely recruited to primarily maximize diversity in gender, age, and socio-economic status. The final 14 participants included 10 women and four men, with two participants under 25 years of age, seven between the ages of 25 and 50, and five over the age of 50. We intended to recruit at minimum 10 CRs and purposely over-recruited to account for potential attrition. Recruitment included hosting stalls in local shopping centers and libraries, physical posters in community locations, sponsoring a local rugby game, handing out flyers, and online posts in local Facebook groups.

**Figure 1:**
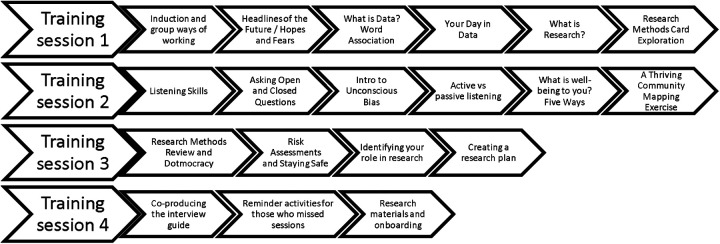
Round ‘Ere community researcher training overview.

CRs were trained over three day-long sessions and one evening session, see Figure 1 for a brief visual outline of the sessions. The training drew on the Clubmoor Toolkit (43), another local place-based PAR and peer research project undertaken within the LCR. This included introductions to quantitative and qualitative research methods as well as practical information including how to collect informed consent, how to conduct an interview, and how to design a recruitment plan. In addition to research training, CRs were guided in exercises in understanding personal bias, what well-being meant to them, and datafication awareness. Datafication awareness drew on exercises around defining and exploring the types of everyday data collected about ourselves created by Our Data Bodies (44). In addition, CRs were asked to design “their day in data”. This was an LCR CDC exercise where CRs identified their day-to-day activities from when they woke up to when they went to bed. They were then asked to think about how those activities could be translated into digital information. This method used a blank piece of paper divided in half where participants listed the activities in their day on the top half of the paper from morning to night and their digital records on the bottom half of the paper. Examples ranged immensely but included smart meter energy data used to track appliance usage, smart phone data collection, and CCTV recordings of traffic. These exercises were used to inspire conversations about how data is currently used in community and how it could be used differently.

As part of the training, CRs voted on the research method they thought would be most appropriate to explore well-being and data with their fellow residents. We first refined the potential options from examples given during the training, then used dotmocracy to vote on the options (selected by CRs), being a survey, semi-structured interviews, focus groups, or photovoice. They chose and helped design a semi-structured interview guide method to collect data from their community. The interview guide included questions on what made individuals feel well, what community wellness meant, and how information or data showed up in their day-to-day lives. There were also additional prompts designed by residents in case people struggled to answer the questions, including a story-prompt, a photo prompt, and examples of data sharing. Over summer 2023, CRs interviewed over 200 of their fellow residents. We then ran three workshops to analyze their results with them and co-design a well-being data hub with local policymakers and voluntary sector representatives. The first workshop focused on initial results from the interviews with CRs only. The second and third workshop included all stakeholders and CRs to discuss the results, the potential usage of a data hub, and case study project preferences. Further details on the methods, the materials used in the project and the results of the CRs interviews are available in the Round ‘Ere report (41).

### 2.2 Research methods and analysis

We utilized repeated, qualitative semi-structured interviews to explore CRs' expectations and experiences of taking part in the project. Interviews were conducted at three time periods: immediately before the training, after the training, and after the CRs completed data collection. All interviews were conducted between May 2023 and September 2023. Sixteen CRs took part in the first interview, 13 in the second, and 13 in the final. One CR who completed the first interview, was unable to complete the training due to time conflicts and left the project. One CR left after completing the training due to disinterest. In total, 14 CRs took part in the project from start to finish. One CR's second interview and a different CR's third interview were not successfully recorded and were lost. In total, 42 interviews were analyzed.

Authors of this article, ER and GP from the research team split the interviews between them, with frequent discussion and refinement of the semi-structured topic guide throughout the three phases. Interviews were held both in-person and online, to the preference of the interviewees. The first topic guide included asking about understanding and perceptions of well-being, data, and the community of Widnes, as well as expectations for the project. The second topic guide also included questions about data and well-being perceptions alongside perceptions of the training and planning for the research project. The third topic guide included questions about results from the CR project and perceptions of the research process and project overall. The topic guides were semi-structured—the interviewers used the questions and topics to prompt CR to think about their experiences of taking part in the project more broadly. We drew specifically on CR statements around their experiences and did not use, for example, their perspectives on well-being in this paper.

We used thematic analysis to explore the benefits and challenges perceived by the CR, along with their expectations and experiences, at each stage of the project (45). Thematic analysis was employed without drawing on any existing coding framework. Rather, we took a collaborative and inductive approach to coding and re-coding themes exploratively. We rooted our analysis in the perspectives of CRs to explore the usefulness of PAR to a PDS project and emphasize the participatory nature of both methods, thus prioritizing the experiences of the CR themselves rather than looking at other stakeholders or other forms of academic analysis. ER and GP analyzed all the interviews in tandem including multiple stages of coding, synthesization, and re-coding until agreement was reached (45). Quotes are written verbatim and indicated by CR and interview number as [CR# I#]. All analysis was conducted using qualitative analysis software NVivo 12 and 13.

## 3 Results

Four main themes emerged from the interviews with CRs on their expectations and experiences: (1) the role of CRs' motivation in taking part and their perceptions of project impact, (2) the role and development of confidence in CRs' perceptions of their own success, (3) the importance of community building through an appreciation of diversity, and (4) the value in developing CR agency by putting participatory process at each stage of the project.

### 3.1 Motivation and perceptions

How CRs described their interest and motivation to take part varied both along the lifetime of the project and between the CRs themselves. They described at times opposing views in their initial motivation to take part ranging from minimal expectations to instrumental hopes for learning to strong community-based altruism.

CRs often described strong altruistic motivations to take part in the project. Indeed, their motivations were at times described as making a better world. They saw the potential for strong community benefit due in part to the project prioritizing community voice as well as the broad definitions of well-being and data taken in the project. These broad definitions allowed CRs to imagine varied and multitude impacts—e.g., improved nightlife from better information about changes over time, better healthcare service provision by improved data sharing to identify need, or a more vibrant economy by identifying information like foot traffic that could help local business growth.

Interviewer: So, as we talked about what we're going to do in the project [around] well-being and information, what do you hope the outcome of the project is?

Interviewee: We can live in a better world. [CR5 I1]

Well, I suppose it's just as you said, really, that if it identifies what are the key things that people are concerned about? And you know, … you can't solve that the housing issue overnight, but maybe things like the park you know. [CR7 I1]

That it improves things for people in general, then we end up with resources that are actually going to be used, rather than what someone thinks we might need which was often [what] has been done in the past. [CR9 I1]

This theme often emerged alongside descriptions of a hopeful nostalgia of their community and a strong pride in place. Widnes was described as formerly prosperous, and the idea of addressing residents' hopes for the future was welcome to improve the current socioeconomic problems impacting the area. In a similar vein, CRs' hopes were tempered by concern that the project would not succeed and not benefit the community at large. As one CR stated, “I don't have any concerns about the project. It's- it's whether it will be influential in the long run.” [CR15 I1] In other words, their motivations to take part were altruistic and hopeful but their confidence in the potential for change varied.

When I grew up in this town, you said to people, I'm proud to come from Widnes..In my words. It's gone to the dogs… there's nothing here for my children. I don't want them to grow [up here] at the minute. So, my outcome of this [project] will be that it will give the town more pride. And it will hopefully make it a little bit better. [CR10 I1]

Nothing's gonna change. That's my big concern. So, collecting all this data, collecting all this information, and then it just festers on a shelf like a book in [a] library. [CR4 I1]

Alongside concepts like altruism, some CRs expressed their motivations more instrumentally as being open or as building skills in research. CRs came with a range of backgrounds and levels of experience in formal learning and research. CRs who had less research experience expressed broader interest in research as a concept and at times open expectations about what the project would offer them.

So yeah. I'd like to understand people in the community better. I'd like to, obviously train to do research properly. [CR10 I1]

Interviewer: And is there anything in the research training we're going to start today that you hope we cover?

Interviewee: No, I'm open book. I'm ready to go with the flow today. [CR16 I1]

Well, I hope to learn how to formulate some of those questions. I suppose would be really interesting how to formulate questions in a way that really get at the heart of what we're trying to … answer, you know. So, asking questions in the right way would be really useful. And then the hope? To just learn some, some analysis would be good if I could learn some basic analysis would be cool because I've not really done any like empirical analysis. [CR1 I1]

Three CRs, who had experiences with postgraduate research, expressed a desire to either reacquaint themselves with research after time away from academia or to learn about methods outside of their previous areas of research. Some had specific hopes on learning more about how to do community-based research—including interview skills and how to formulate research questions.

But so, I'm hoping to get an idea of what it's like to work with people. For one, because that's a skill I can take with me when I do come to complete my PhD. So that's a big one. For me, working with people and working with part of working with people is things like recording interviews, I've never had to do that for my, my dissertations or anything before. [CR6 I1]

### 3.2 Confidence and success

Confidence was described as key for CRs to perceive and evaluate both their own success and the success of the project overall. All CRs described the training, workshops, and research process as an overall positive experience. This included enjoying the process of training itself, the opportunities to interact with a range of people in their local area and overcoming the challenge of conducting research in their community.

Interviewer: And would you consider doing something like this again?

Interviewee: Absolutely. Absolutely. I -I actually really enjoyed it. So, I think -I think I would do it again. [CR2 I3]

These positive experiences were often connected to how confident each CR felt to take part in the project. A few CRs expressed from the first interview a strong sense of confidence in talking to their community which was connected to feeling that they would succeed. For example, one CR stated “I deal with the public all the time. So, I've no issues about putting anybody in an uncomfortable situation, I'll be self-confident about going forward.” [CR3 I2] Several CR, however, were unsure about their ability to take part due to a lack of familiarity with research and data. Subsequently, the training workshops were key to building confidence to succeed. CRs described the value of training in broadening their understanding of research, even amongst the CRs who were more familiar with research methods.

Yeah, it [the training] was really useful. It it helped me to overcome my fear…I was always afraid to to reach people I didn't know myself. [CR14 I3]

It's [a bit] out my comfort zone… when I got stopped at the Widnes market to ask about it. If they told me [it] involved all this. I don't know, that [I] would have done it…And it's just, I don't -don't feel confident in myself, but I have enjoyed it and feel like I've been comfort[able] so. — It was just my confidence. Nothing else. [CR8 I3]

So, I didn't really know what to expect…But the style of the question, and how it's gonna be collected at the way that was explained, and the training was really expansive. And it's broadened the whole spectrum…as far as really about what's going to be carried out. [CR3 I2]

The process of research, it's not just going out and asking questions or doing interviews, it's the transcribing. It’s the work behind it. The ethics, like that overall view of research. It's just broadened for me. [CR10 I2]

In particular, CRs enjoyed the range of methods presented including narrative, survey, and creative social sciences methods. The interactive format of the training was described as bringing together academics and local people in an informal setting that otherwise would not happen.

I have an okay understanding of research, [what] surprised me a little bit, but I liked, was that you're holding all the research methods, the cards and looked at them all you didn't just say, well, these most rare Let's go. Yeah. So even though some are quite far out, and they will be used, but people still got to know about, and I did like that because obviously it gives people kind of space to say, well, tell me more about it. You know, and I think that's really good. [CR6 I2]

The training itself, I've- I've fully enjoyed it. I like getting into it. I loved hearing other people… I -I especially really, I don't know what it was about [the academic lead], but he absolutely fascinated me, and I loved speaking to him. [CR10 I3]

As for the research process itself, several CRs described a journey from feeling at first wary and unsure to get started that then built into a sense of confidence once they felt more familiar with the process of conducting the interviews. Several CRs also wished to continue the process of research and expressed disappointment at the just over one-month timescale of the data collection phase. Confidence to conduct research was often described both as confidence in key skills like interviewing and confidence to adapt their research plan and strategies.

I had a moment of panic. But when I thought about it, you know? You've given us the training you'd guided us in the right direction…that's about wanting it to be right, I think. [CR11 I3]

I got four [interviews] which is nowhere near what I wanted … It made me a little bit sad cause I could feel my confidence growing…It was a learning curve already for me. Like I learned a lot just from doing those four. That it could -could have been so much more impactful if that makes sense but I really enjoyed it. [CR6 I3]

I suppose with practice you feel a bit more confident, don't you, about trying to prompt people, but without putting words in their mouths. So, I felt it went so long it it got easier in that sense. Yeah, sort of learn ways of actually encouraging people to say things. [CR7 I3]

CRs also valued developing a critical understanding of how to revise an interview guide in the light of their experiences of fieldwork. This included confidence to adjust questions slightly, change the order of questions, and how to introduce the questions. CRs described this as wanting to get the questions right to ensure both the success of their interviews and the validity of the results. This included a reflection from some CRs that asking about well-being was at times easier than asking about data. They felt the need to provide explanation for the logical shift from wellness to information.

I found that it worked really well because we were able to kind of go off script and really try to tease out what sometimes what people were saying. [CR1 I3]

Some CRs also experienced challenges during the research process that decreased their confidence to succeed, including worries about recruitment. For example, several researchers expressed frustrations that they would not be able to find enough interview participants and discussed varying how they recruited interviewees to address this. CRs also expressed frustration that individuals who agreed to take part did not always follow through to taking part in an interview.

And another thing that surprised me…the amount of people that just don't want to do it. Or they say they will, and they waste your time a little bit or not intentionally. But you know, they say I'll do it. They'll even exchange loads of messages with you. And they'll say, OK, so when and then you try to pin them down to something specific and then they just go off radar and I don't know if they get any kick out to that? [CR6 I3]

### 3.3 Community-building and diversity

CRs also described their perception of the value of a participatory action research process as enabling community building and diversity. Inclusivity and the ability to connect to each other and the community was a common theme in discussions with CRs about their experiences.

But what I -what I've learned now is that it's probably still going to be a bit scary, but it's not something you do on your own. So -So in all the times I thought about it, I thought of me going out there being a lone soldier, if you like. And really, it's nothing like that. I said, well, this project certainly isn't, and I imagined my a lot of projects work along similar lines at least. So, you're not you're not on your own… Now I see it more as a community thing rather than a clinical thing … So, I think that's what I've learned that -that you can research with people, and it can be friendly, you know, friendly, but you have your backup and your team, basically so so it's not just you against the world. [CR6 I2]

In both the training and the research process, CRs valued opportunities to interact with people outside of their immediate friends and family. As one CR put it “there was a good cross section of different people.” [CR5 I2] This included the opportunity to interact with people who did not align with their own demographic characteristics. The most visible of these being age and race.

But I really enjoyed the group. And it seems like a nice bunch people. And it's interesting to hear other people's thoughts on what we're doing. So yeah, I enjoyed it. [CR11 I2]

The most important I think is … to meet the other people. Yeah. And I think that actually setting up that Whatsapp group, I think it's a really good idea for us to to be able to communicate. [CR7 I2]

This theme also carried over in discussions on the diversity of the participants they were interviewing in their fieldwork. CRs wanted to maximize diversity as much as possible to accurately reflect their community and, in their view, to minimize bias and improve the validity of their results.

So, my current thoughts are the, you know, largely, because you've said it's okay, we can ask family and friends. And that seems the obvious because they are our circle. And it's like, easy. But…it’s still quite close, you know, quite closed. So, I'd like to definitely do a mixture of those things. Obviously, for me, the easiest thing is family and friends. But I definitely like to go out into the community as well. [CR12 I2]

CRs reflected on how to expand recruitment during fieldwork by changing where and how they recruited participants. In particular, CRs wanted to expand the involvement of younger people, communities of color, and non-English speaking communities. While those groups were represented amongst the CRs themselves, many of these groups were perceived to be different to the historical profile of people in Widnes.

I know we've got Polish community here and I -I don't know much else because I've not researched it too thoroughly, but I liked the idea of it seems more diverse when you're walking in the streets like the community, it's not so white as it used to be, thank goodness and. And so, I liked the idea of that. [CR12 I3]

I tried …to find a variety of different interviewees from different educational backgrounds, different ages, different occupations, different interests. [CR9 I3]

Well, obviously, the community it’s an opportunity for people to say what they feel….If we could do something with young people to get their voice heard. [CR7 I2]

### 3.4 Agency and participatory process

A final but essential aspect of the project that CRs reflected on was agency and its association with participatory process. CRs described agency and representation as lacking in existing service provision in the region. They wanted the project to highlight voices from the community. This connected strongly with the common initial desire to take part driven by altruism but moved beyond that to reflect CRs desire for enhanced community voice as a goal in and of itself. One CR described this as creating a ripple effect that would trigger informal conversations about well-being and Widnes within the community, highlighting an unintended impact of research beyond any ultimate research results or data hub.

I hope the people I speak to at least we will get them thinking more about well-being, and then hopefully, have conversations with other people around that as well. So that you can have a bit of a ripple effect of people just thinking about what well-being means more in our community. [CR1 I2]

So, I think, with this project, my understanding is that it will basically allow ordinary people to sort of see themselves reflected back at themselves, when we sort of see NHS and look government policies regarding health care and behavior. [CR13 I1]

CRs were highly supportive of the participatory elements of the project design. The training included several participatory elements, both deciding on the research method and on the interview guide. CRs reflected that they assumed the research method and design would be finalized before the training and were pleasantly surprised by how open and interactive the sessions were.

It was better than what I thought would happen. It was kind of like going back to training that used to happen about 20 years ago…You felt really comfortable and valued and like your opinions mattered and -and the whole ethos. [CR9 I2]

It was a bit nerve wracking at the end because I ran the risk of being the last person- that was close. I had to run up and quickly put one on just [so I] wasn't last. But what I liked about it was everybody did kind of get their say whether it worked out in their favor or not. You know it was done fairly. So, you you've really taken on board the voices of everybody who you working with, it's not just kind of okay we we want you to go out into this, this and this and this you're involved in the process from step one. [CR6 I2]

Participation was valued both as a goal of itself, i.e., representing voice, but also as a means to valuing CRs' time and embedded community knowledge. CRs described this as feeling welcomed, comfortable, and heard in the training process.

So, I thought you made a good job of making sure everyone felt heard, even if you ultimately didn't take on a particular thing that had been said, you know, you'd listened to it and filtered the commission. So, I thought you did that? Yeah…. And we all felt -I felt, you know, really sort of welcomed, and that you'd made a big effort to have an exit to make a nice environment and to make us feel you know … part of the project. [CR12 I2]

## 4 Discussion

The results illustrate that taking a PAR approach to the design of a PDS project around well-being and data shows potential for addressing power and participation in the social justice aspects of datafication, i.e., data justice, through engaging local communities, developing their research skills, confidence and agency, and designing a project on data that can empower community voice. Overall, the project evidenced the value of applying a PAR methodology to a PDS project for both CRs and wider community stakeholders. CRs found the process of participating in the project enjoyable and meaningful for their community. We discuss below how successful our PAR approach was in addressing issues of participation and power in data system design and wider narratives of data justice and datafication for PDS projects.

A key evaluation metric of the success of deliberative and participatory methods is the participants themselves feeling heard and represented through the project (3, 4, 46). In the context of our PAR and PDS project, this relates to whether participants felt empowered and valued in the PAR process itself and as part of the wider data hub project (4, 47). Our CRs' reflections on the process of taking part in the project suggest that CRs valued and were supportive of working alongside the research team and having their opinions and voices prioritized in the project design. CRs described a strong sense of ownership of the process and outcomes due to the multiple ways they were asked to make decisions alongside the formal research team, a key feature of a PAR design. Inasmuch as a goal of PDS is the democratizing of who has a say in data systems, CRs' perceptions of their own agency and involvement in the project evidences the value of taking a PAR approach to PDS.

In addition, the two-phased nature of PAR, i.e., community were represented both as CRs and as interviewees, helped us to address the breadth of who was able to have an impact on data processes, in our case the kinds of data preferred to be used and the ultimate design of a well-being data hub. Our CRs' concerns around heterogeneity of the interviews, and how representative their interviews would be, demonstrates how our PAR methodology brought this issue to the forefront. While this is a single case study of a PAR/PDS project, this suggests potential in how PAR allows CRs, and PDS projects more broadly, time to engage with deeper issues like how to diversify recruitment in a tangible way. We enabled this through the design of the project, especially in leading the CRs through reflecting on recruitment and bias in the training exercises. We also enabled this through our own recruitment process—spending time in person in community spaces that were frequented by different kinds of communities. While this requires significant investment (both in person-hours from the research team and the CRs), it shows the value of this PAR approach in empowering community and democratizing the involvement of citizens in the development of a data technology at the community-level. The core aim of PDS is to expand who has a say in datafied society and, while many participatory and deliberative processes are often critiqued for only representing those who are already in relative positions of social power (48), we found that our PAR methodology, where CRs held decision-making power on recruitment and design of the PDS project, was helpful in addressing these challenges.

A secondary key factor for addressing the success of participatory and deliberative processes around data is impact. Specifically, it is important to consider whether our PAR process addressed the feasibility of creating change in the landscape of datafication and disempowerment at the community level. This means exploring how our PAR and PDS process can promote data justice through challenging data science hegemonies by expanding community power and contextual knowledge from community in data systems in practice (20). We break this concept into two components of datafication that we feel our project was able to address—(1) opportunities for raising individual and community awareness of datafication within the community, and (2) impacting the use of data to make decisions within a community setting.

An important building block for our project, and indeed any PDS project, is surfacing the impact of data on community for participants who are taking part (3, 24, 36). Improving CRs' data literacy was not a specific aim of this case study. That is, we did not specifically design the project to improve the skills and knowledge required to access, manage, and use data (49, 50). However, through the nature of the training sessions and project aims, we did lead the CRs through a critical exploration of what data could (and could not) address in a community setting. This is something that warrants further attention and that future research on PDS and PAR should consider more closely. Data was an explicit point of discussion. However, as evidenced by the motivations our CRs had for taking part, no one came to the project with the explicit purpose of challenging datafication or promoting data justice. Rather, they approached the project to build research skills and benefit their community. This meant that our CRs needed significant guiding in connecting the concept of well-being to the impact of data in community. This is not inherently problematic. CRs were not expected, nor is the UK population at large due to relatively low levels of (critical) data literacy (50), to join the project as data activists. As highlighted in other community data projects like Measuring Wellness, community altruism or concern is a powerful motivator for getting individuals involved in discussing measurement and data (33). In addition, Kennedy (24) highlights the importance of attending to the everyday lived realities of non-experts in understanding the impacts of datafication and promoting data activism rather than solely the larger narratives of moral imperatives or harm from datafication. Our project adds to this literature. While the Round ‘Ere case study was explicitly about data and well-being, our CRs would likely not have described it as such. Indeed, their assessment of impact and project success was more connected to their own perceptions of confidence and community change than democratization of data. Building on Kennedy's work (24), we argue this is a contextual (and implicit) form of “everyday” data activism.

We highlight in the literature review how existing community data projects engage with implicit and explicit topics—e.g., wellness and data or transport and data. This project took a similar approach—being both about well-being and data. This reflects both the nature of data and of the wider narrative of datafication. In other words, the impacts of data and datafication are defined by what they are representing and impacting respectively. A necessary step to defining our PAR project and motivating CRs to take part is defining what *action* we wish to take, and therefore what data meant and how it could be stewarded towards community benefit. This kind of “everyday” data activism is then explicitly about stewarding data towards positive outcomes through concepts of well-being. We found our CRs were less concerned with labelling the project as relating to data or well-being and more concerned with what positive change looked like for them. In short, their activism was focused on community change but stewarded *by* data. The PAR method allowed CRs, and the wider research team, to hold these dual motivations. It was meaningful to look at issues of PDS through a PAR lens as it reflected and amplified needs and priorities of the community of interest—it allowed space for their own perceptions of impact. Thus, the PAR methodology showed evidence of success both in expanding the diversity and impact of community on how to use data, including in this case the design of a data hub, but also in fleshing out the importance of broader social and community justice in the use and design of data systems themselves.

The next stages of the Round ‘Ere project as described in the Methods section above will help further inform the impact of a PAR approach to PDS in the wider narratives of data justice, social justice, and datafication. The altruistic motivations of our CRs highlight both a benefit, as described above, but also a challenge. Their expectations of addressing housing, youth crime, poverty, the economy etc. will sit in conflict with what this project can realistically achieve. Our process of calling community in to design the kinds of data they want to measure will need to result in measurable change to ultimately be considered a valid and helpful process. This also highlights the importance of considering when we interviewed the participants. While we collected their experiences during the project—evaluating how successful this method will be in the long run will require a longer-term approach to assess community perspectives and perceptions over time. While we argue in this paper that PAR is a useful methodology to explore questions of empowerment and whose voices matter in how data is used at a community level, any social change will only be valuable as long as it is also demonstrable. Future research will need to build on this study to explore the conditions for the impact of PAR and PDS on the use of data in community in this context and others.

### 4.1 Limitations and reflections

Round ‘Ere explored the adaptation of PAR to PDS and was a first step for the LCR CDC in designing community data tools and projects. This paper primarily focuses on the intersection and usefulness of a joint PAR and PDS approach. Further work based on the project will address the role of data literacy in PAR and PDS as well as the design of community data projects using the PAR and PDS results.

Limitations of this study include the lack of additional evaluation, and time. This paper is explorative, interdisciplinary, and descriptive due its focus on methodology. More specifically, we drew on varied literatures without relying exclusively on one single theoretical perspective, while our focus on practice allowed us to grapple with issues of feasibility. Even though the interviews were essential in understanding the CR perspective, further work will need to address the usefulness of this approach to other communities and individuals involved in PAR and PDS, including local governments, digital and tech developers, and policymakers. To further build an understanding of PAR and PDS, this work will need to be trialed in different communities and contexts. Equally, from a participatory methods perspective, further evaluation of the project will be valuable in comparing its stated aims to its ultimate impacts. In particular, questions that may be addressed in the future include how the experiences of CRs vary by community identity and demographic background, which was not explored in this study.

Finally, the nature of research and research grant funding mean that an academic group launching a PDS project and studying its methods will never be a sustainable process for community improvement. Funding ends, researchers move on, but community remains. For PDS to be a sustainable process, work like this should be sponsored and led by government and community respectively. The researchers on this project felt at times the burden of responsibility far outweighed a typical research project, and that responsibility must be necessarily shared between stakeholder groups to begin to address the kinds of challenges highlighted. However, part of opening a conversation and surfacing community data justice means addressing this responsibility.

## 5 Conclusion

In this paper, we present results from a PAR and PDS project called Round ‘Ere conducted in the UK town Widnes. Members of the community were trained in social science research methods to collect data from members of their own community about their perceptions of well-being and the role of data in making decisions to improve their community. This article addresses a gap in the literature on studying how to operationalize PDS in a local community setting. Through semi-structured interviews, we explored the feasibility of taking a PAR approach to PDS. CRs valued the project's participatory process, diversity, and opportunities for confidence-building. They came to the project with strong motivations for community change, which was at times challenging within the scope of a PDS and PAR project. The results illustrate that taking a PAR approach to the design of a PDS project around well-being and data shows potential for problematizing datafication at the everyday and community level. Future research should build on this study to further explore the conditions and contextual factors for successful PAR in the context of PDS projects, and how to evaluate positive and sustainable impact on community projects related to datafication and data justice.

## Data Availability

The datasets presented in this article are not readily available because once anonymized and the project is closed, interview data will be available through the University of Liverpool at: https://datacat.liverpool.ac.uk/. Requests to access the data can be directed to the corresponding author.
